# Determining the role of statins in Parkinson's disease risk reduction and disease modification: A comprehensive meta‐analysis of 4 million participants' data

**DOI:** 10.1111/cns.14888

**Published:** 2024-08-04

**Authors:** Abdelrahman Mady, Yehia Nabil, Asma Daoud, Asmaa Zakria Alnajjar, Taleb Alsalloum, Menna Marwan, Maickel Abdelmeseh, Moaz Elsayed, Abdulrahman Krayim, Mohamed Alaa, Mahmoud Masoud, Nagham Bushara, Roaa Faisal, Hayat Ahmed, Mohamed Saad, Zineddine Belabaci, Matthew J. Barrett, Brian Berman, Ahmed Negida

**Affiliations:** ^1^ Medical research group of Egypt, Negida Academy Arlington Massachusetts USA; ^2^ Faculty of Medicine Al‐Azhar University Cairo Egypt; ^3^ Faculty of Medicine Zagazig University Zagazig Egypt; ^4^ Medical research group of Algeria Negida Academy Arlington Massachusetts USA; ^5^ Faculty of Medicine Ferhat Abbas University Setif Algeria; ^6^ Medical research group of Palestine Negida Academy Arlington Massachusetts USA; ^7^ Faculty of Medicine Al‐Azhar University Gaza Palestine; ^8^ Medical research group of Syria Negida Academy Arlington Massachusetts USA; ^9^ Faculty of Medicine University of Hama Hama Syria; ^10^ Faculty of Medicine Port Said University Port Said Egypt; ^11^ Faculty of Medicine Alexandria University Alexandria Egypt; ^12^ Faculty of Medicine Cairo University Cairo Egypt; ^13^ Faculty of Medicine Minia University Minia Egypt; ^14^ Faculty of Medicine Al‐Azhar University New‐Damietta Egypt; ^15^ Medical research group of Sudan Negida Academy Arlington Massachusetts USA; ^16^ School of Medicine, Ahfad University for Women Omdurman Sudan; ^17^ Faculty of Medicine Azza University College for Women Khartoum Sudan; ^18^ Faculty of Medicine Ain Shams University Cairo Egypt; ^19^ Faculty of Medicine Djillali Liabes University Sidi Bel Abbes Algeria; ^20^ Department of Neurology Virginia Commonwealth University Richmond Virginia USA

**Keywords:** disease risk, hydroxymethylglutaryl‐coenzyme A reductase inhibitors, meta‐analysis, Parkinson's disease, statins, symptom progression

## Abstract

**Background:**

Many observational studies have examined the association between statins and the incidence of Parkinson's disease (PD) in high‐risk populations. On the other hand, clinical trials as well as other observational studies investigated the safety and efficacy of statins in slowing disease progression in PD patients. However, the evidence has been inconclusive in both questions. To that end, we conducted this systematic review and meta‐analysis to synthesize evidence on the role of statins in decreasing the risk of PD among high‐risk populations and as a possible disease‐modifying agent for patients with PD.

**Methods:**

A comprehensive literature search of electronic databases including PubMed, Scopus, Cochrane, and Web of Science has been performed. Relevant studies were chosen and data were extracted and analyzed using RevMan software version 5.4.1.

**Results:**

Twenty‐five studies (14 cohort, 9 case–control, and 2 randomized controlled trials) have been included in the present systematic review. Of them, 21 studies reported the association between statins and PD risk. Statins were found to significantly reduce the risk of developing PD (pooled RR 0.86, 95% CI [0.77–0.95], *p* < 0.005). Four studies investigated statins as a disease‐modifying agent. The pooled mean difference (MD) in the UPDRS‐III from baseline to endpoint did not differ significantly between the statin and control groups (MD −1.34 points, 95% CI [−3.81 to 1.14], *p* = 0.29).

**Conclusion:**

Although epidemiological observational studies showed that statin use was associated with a reduced risk of PD, current evidence is insufficient to support the role of statins in slowing the progression of PD. These findings are limited by the fact that most of the included studies are observational studies which carry a high risk of confounding bias which highlights the need for future well‐designed RCTs.

## Graphical abstract

A systematic review and meta‐analysis of published literature. Data from 21 observational studies showed that individuals who received statins in the past had lower risk of developing PD. However, four prospective studies supplementing statins for PD patients showed no evidence of benefit.

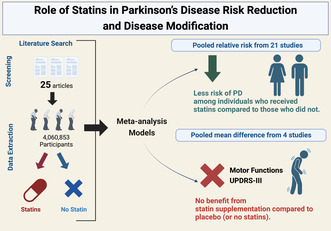

## 1 INTRODUCTION

Parkinson's disease (PD) is a chronic, progressive neurodegenerative disorder affecting approximately 1% of the population over 60.^1^ It is characterized by the loss of dopamine‐producing neurons in the substantia nigra region of the brain, causing motor and non‐motor symptoms, such as tremors, rigidity, bradykinesia, postural instability, cognitive impairment, and depression.^2^ The exact cause of PD is unknown; however, neuroinflammation and microglial activation are among the key pathological processes involved in the disease.^3^ Despite the extensive research over the past decades, current treatment options for PD are mainly symptomatic with no drugs have been found to prevent, slow down, or cure PD.

A simple and rapid method of therapeutic development is to screen existing drugs for potential benefits for the underlying disease. Statins are a class of medications commonly used in lowering cholesterol levels. Recent studies have shown that statins have anti‐inflammatory properties that may play a role in the progression and treatment of PD.^4^ This was further corroborated by some epidemiological studies reporting a significant association between statins and the reduced risk of PD. However, this evidence is conflicting and not conclusive. For example, a prospective study in 2015 showed that statin use was associated with significantly higher PD risk (OD 2.39, 95% CI [1.11–5.13])^5^ while another study in 2016 found that statin use was linked to lower risk of PD (HR 0.65, 95% CI [0.57–0.74] in females and 0.60 95% CI [0.51–0.69] in males).^6^

Subsequent clinical trials investigated the potential disease‐modification effects of statins in PD. A randomized controlled trial (RCT) in 2021 found a beneficial effect of lovastatin in reducing the mean UPDRS motor score compared to placebo (MD −3.18 vs. −0.50; *p* = 0.14).^7^ On the other hand, another RCT in 2022 found that simvastatin was associated with deterioration in the UPDRS III score compared with the placebo group (1.52 points).^8^

Seven previous systematic reviews and meta‐analyses evaluated the association between statins and PD.^9^, ^10^, ^11^, ^12^, ^13^, ^14^, ^15^ However, their inclusion criteria were limited only to observational studies and the only outcome of interest was the risk of PD development in high‐risk populations.

We conducted this systematic review and meta‐analysis to synthesize evidence that answers two research questions: (1) is statin use associated with a lower risk of PD in high‐risk populations? And (2) does statin supplementation slow the progress of PD compared to placebo?

## 2 METHODS

We followed the Preferred Reporting Items for Systematic Reviews and Meta‐Analyses (PRISMA) statement guidelines for reporting this systematic review and meta‐analysis.^16^ The protocol of this study was prospectively registered in PROSPERO (CRD42023396879).

### 2.1 Inclusion and exclusion criteria

When selecting studies for this review, eligibility criteria were defined using the participants, interventions, comparisons, outcomes, and study design (the PICOS model).

For the first research question, we included studies where (1) the population was patients with PD, (2) the intervention was statins, (3) the comparator was normal controls, (4) the outcome was the risk of PD occurrence, and (5) the study design was either cohort or case–control.

For the second research question, studies that meet the following criteria were included: studies where (1) the population was patients with PD, (2) the intervention was statins, (3) the comparator was normal controls, (4) the outcome was the changes in Unified Parkinson's Disease Rating Scale—Part III (UPDRS‐III), and (5) the study design was RCT or observational study.

Studies that meet the following criteria were excluded: (1) basic science and preclinical laboratory studies that did not involve any clinical data, (2) publications that were case report, case series, or literature review, and (3) studies in languages other than the English language.

### 2.2 Search strategy and keywords

A comprehensive internet search of electronic databases including PubMed, Scopus, Cochrane, and Web of Science has been performed until February 2023. A specific set of keywords and search strategies were used to ensure that the results were relevant. The keywords used were “statin(s),” “HMG‐CoA reductase inhibitor(s),” “simvastatin,” “atorvastatin,” “pravastatin,” “fluvastatin,” “rosuvastatin,” “lovastatin,” “Parkinson's disease,” and “PD.” The keywords were combined with each other using the Boolean operators “AND” and “OR.” Additional records were identified through previous systematic reviews and a manual search. All electronic records were exported to Rayyan software to conduct the screening process.^17^

### 2.3 Selection of studies

To ensure the selection of relevant studies in our research project, a two‐phase screening process was conducted with the participation of all authors. During the first phase, two groups of authors independently assessed the eligibility of the title and abstract of each article, and this process was repeated twice to ensure accuracy. In the second phase, the full text of all articles that passed the first phase was examined to identify potentially included studies for our research project.

### 2.4 Data extraction

A data extraction excel sheet that was accessible to all authors was created. All authors participated in data extraction. Extracted data for each study included: study ID (last name of first author and the publication year), country, study design, length of follow‐up, the total number of participants, study population, number of PD patients, outcome measures, and key findings.

### 2.5 Assessment of risk of bias in included studies

We used the Newcastle–Ottawa Scale (NOS) to assess the risk of bias in the included observational studies.^18^ The NOS is a tool specifically designed to evaluate the quality of observational studies and consists of eight items grouped into three domains. These domains include the selection of study participants (four points), comparability between groups (two points), and ascertainment of exposure (in case–control studies) or outcomes (in cohort studies) (three points). The maximum score of nine points indicates the least risk of bias. Based on the total score, we categorized the studies into low, medium, or high risk of bias, with scores of ≥9, 7–8, and <7, respectively.

Additionally, we used the Cochrane risk of bias‐2 (ROB 2) tool to assess the risk of bias in the included RCTs.^19^ This tool evaluates several aspects of the RCTs, including sequence creation, allocation concealment, blinding, inadequate outcome data, selective outcome reporting, and other potential sources of bias.

### 2.6 Measures of drug effect and statistical analysis

We conducted this systematic review and meta‐analysis to synthesize evidence that answers two research questions: (1) is statin use associated with lower risk of PD in high‐risk populations? And (2) does statin supplementation slow the progress of the disease compared to placebo?

For the first research question, the primary outcome measure was PD risk. It is measured as odds ratio (OR), risk ratio (RR), or hazard ratio (HR). OR represents the odds of using statins in the past compared between the PD patients and non‐PD controls. An RR greater than one indicates an increased risk of PD and an RR less than 1 indicates a decreased risk of PD. All effect sizes represented as RR, OR, and HR were included and treated the same because of the low incidence of PD.^20^

For the second research question, the primary outcome measure was motor functions measured by the UPDRS‐III score. This continuous outcome was expressed as the mean difference (MD) between the study groups in terms of the changes from the baseline. The MDs and their corresponding 95% CIs were pooled in the meta‐analysis models.

All the effect sizes were pooled in a meta‐analysis model using the RevMan software version 5.4.1. A random‐effects meta‐analysis model, using DerSimonian–Laird method, was employed to calculate the final pooled effect estimate. This model was chosen over the fixed‐effects model to account for withing‐study and between‐study variability. Following the main analysis, we preplanned for subgroup analyses according to study design, location, adjustment for age, adjustment for sex, adjustment for smoking, and statin type.

In case of missing data, we contacted the corresponding authors to request them. Second, in case of nonresponse, for studies that reported only pretreatment and posttreatment means, the mean change was calculated by subtracting baseline (pretreatment) from final (posttreatment) values and standard deviations of change were imputed according to Cochrane recommendations using the equation below with a conservative correlation coefficient (*r* = 0.5).^19^

$$\sqrt{SD^{2}\text{baseline}+SD^{2}\text{final}-\left(2\times r\times \mathrm{SD}\text{baseline}\times \mathrm{SD}\text{final}\right)}$$

Other missing data were imputed using the available data, for example, confidence interval, *p*‐value, *t*‐value, and standard error, according to Cochrane Handbook recommendations. For the statistical analysis, we used the Review Manager version 5.4.1 for Windows.

### 2.7 Assessment of heterogeneity

First, heterogeneity was inspected visually in the forest plots by looking for the absence of any common overlapping point among all the pooled 95% confidence intervals. Statistical heterogeneity was assessed using the chi‐squared test, which tests the chi‐squared distribution of the Cochrane *Q* test. We used the I‐squared test to quantify the amount of variation among studies caused by factors other than chance. I‐squared values were interpreted as low (25%), moderate (50%), and high (75%).^19^

### 2.8 Publication bias

Publication bias was assessed by visual inspection of funnel plots of the meta‐analysis models. The existence of asymmetrical distribution of the studies around the pooled effect estimate indicates the possibility of publication bias. According to Sterne et al.,^21^ funnel plot asymmetry should not be used as a tool to assess the publication bias unless there are at least 10 studies in the meta‐analysis. If case of less than 10 studies, the power of the test is too low to distinguish chance from real asymmetry.

## 3 RESULTS

### 3.1 Search results

**FIGURE 1:**
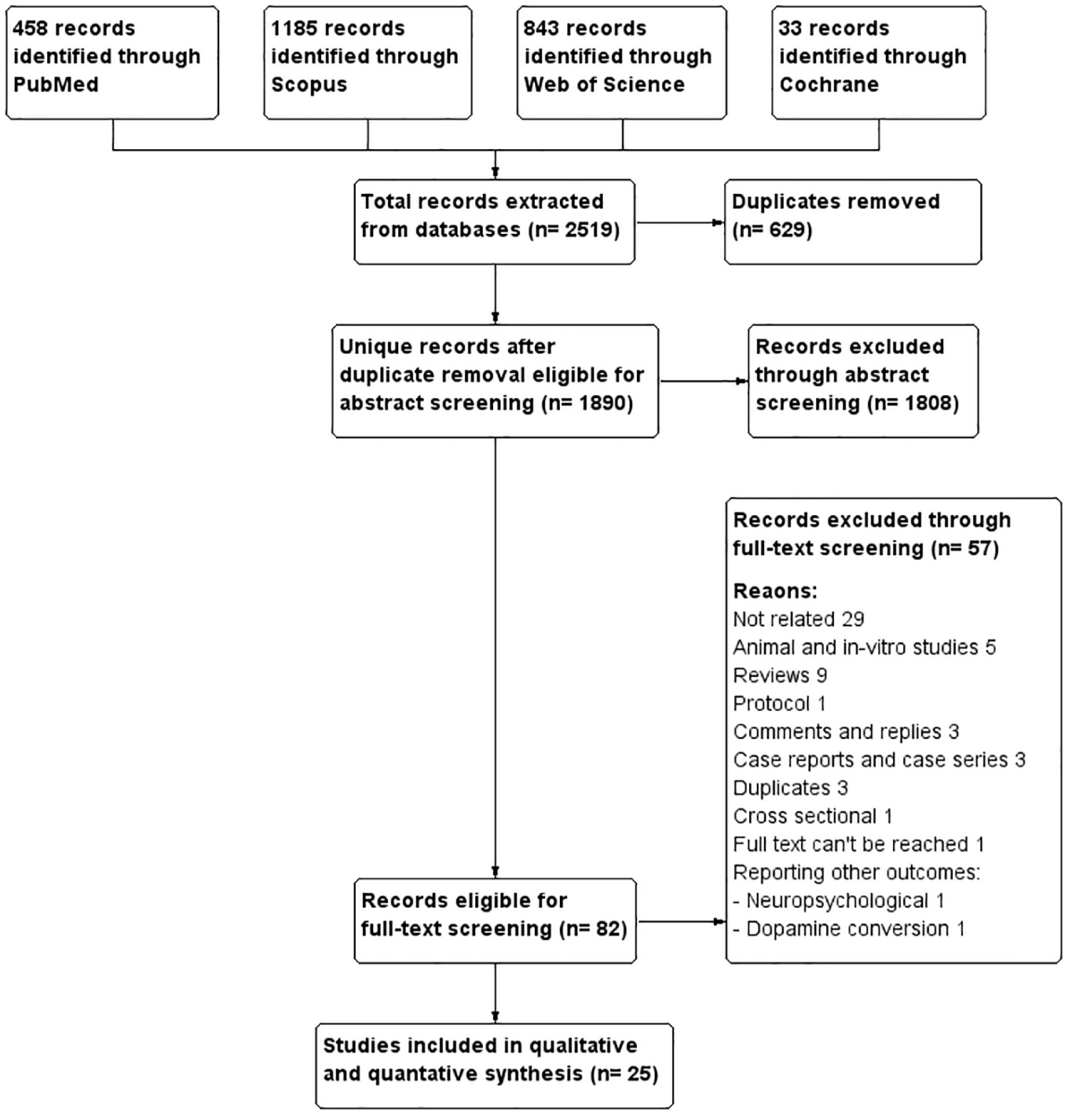
PRISMA flow diagram showing the details of the study selection process.

We identified 2519 references through computerized and manual searches, removed 629 duplicates, and screened the titles and abstracts of the remaining 1890 records. We excluded 1808 records after reviewing the titles and abstracts because they did not meet the prespecified inclusion criteria. The full texts of the remaining 82 studies were obtained and assessed again according to the eligibility criteria. This time, we excluded 57 studies because they were either animal experiments, in vitro experiments, review articles, case reports, comments, replies, irrelevant studies, or meeting abstracts, leaving 82 records. Finally, 25 studies including 23 observational studies and 2 RCTs fulfilled our inclusion criteria and were included in the meta‐analyses (Figure 1). This includes 21 studies addressing the first research question (Does statin reduce the risk of PD in high‐risk populations?) and four studies addressing the second research question (Among patients with PD, does statin supplementation slow the progress of PD?).

### 3.2 Study characteristics

The present systematic review included 25 studies with 17 years as a span of publication time of the articles starting from 2006 and ending in 2023. Among the included studies, 23 were observational (14 cohort and 9 case–control), and 2 were RCTs.^7^, ^8^ Nine of the studies were conducted in the United States,^5^, ^22^, ^23^, ^24^, ^25^, ^26^, ^27^, ^28^, ^29^ three in the United Kingdom,^8^, ^30^, ^31^ two in France,^32^, ^33^ two in Israel,^34^, ^35^ two in China,^6^, ^36^ two in Taiwan,^7^, ^37^ one in South Korea,^38^ one in Italy,^39^ one in the Netherlands,^40^ one in Denmark,^41^ and one in Canada.^42^ The total number of participants included in all studies was 4,071,794, including 35,283 PD cases. Among the 23 observational studies, statin use was detected by reviewing medical records in 6 articles, databases in 13 studies, and self‐reporting in 4 studies.

### 3.3 Methodological quality

ِAfter applying NOS to the 23 included studies, we categorized the studies into high, medium, or low quality, with scores of 9, 7–8, and <7, respectively. Two studies were found to be of high quality,^30^, ^36^ 15 studies were found to be of medium quality,^5^, ^6^, ^24^, ^25^, ^26^, ^27^, ^28^, ^29^, ^33^, ^34^, ^37^, ^38^, ^39^, ^40^, ^41^ and 6 studies were found to be of low quality.^5^, ^23^, ^31^, ^32^, ^35^, ^42^ The NOS score of each study is shown in Table 1.

**TABLE 1:** Characteristics of the included studies.

Study ID (first author + publication year)	Study duration	Study type	Population	PD Cases	Definition of statin use	Country	Variables adjusted	(NOS) Score
De Lau 2006	1990–2004	Co	6465	87	Medical record	Netherlands	1, 2, 7	8
Huang 2007	2002–2004	C‐C	236	124	Medical record	United States	1, 2, 7, 19	7
Wolozin 2007	2003–2005	Co	1,226,198	5107	Database	United States	1, 9, 11, 12, 14, 16, 19	6
Becker 2008	1994–2005	C‐C	7274	3637	Medical record	United Kingdom	7, 8, 10, 11, 15, 16, 18, 19	9
Samii 2008	1997–2003	C‐C	23,780	4756	Medical record	Canada	2, 18, 19	6
Wahner 2008	2001–2007	C‐C	654	312	Self‐report	United States	1–5, 7	7
Mutez 2009	2004–2006	Co	390	390	Database	France		3
HippisleyCox 2010	2002–2008	Co	2,004,692	3553	Database	United Kingdom	1, 7, 8, 17, 19	8
Ritz 2010	2001–2006	C‐C	11,582	1931	Medical record	Denmark	1, 2, 9, 10	7
Gao 2012	1994–2006	Co	129,006	644	Self‐report	United States	1, 7, 8, 11, 13, 14, 16, 19, 20–23	7
Friedman 2013	2000–2007	Co	87,971	824	Database	Israel	1, 2, 6, 7, 11, 14–17	8
Lee 2013	2001–2008	Co	43,810	1886	Database	China	1, 2, 11, 14–16, 19	9
Huang 2015	1987–2008	Co	15,296	56	Medical record	United States	1–3, 7, 9, 11, 16, 19, 20, 25	6
Lin 2016	1996–2008	Co	50,432	651	Database	China	1, 2, 6, 9, 11, 14, 15, 26	8
Shalaby 2016	2009–2014	C‐C	230	108	Self‐report	United States	1, 2, 5, 7, 8, 16	7
Zhang 2017	2017–2017	C‐C	161	91	Self‐report	United States	1, 2, 7, 19, 25	8
Rozani 2017	1999–2012	Co	232,877	2550	Database	Israel		6
Liu 2017	2008–2012	C‐C	4644	2322	Database	United States	18, 19	7
Jeong 2019	2002–2015	Co	76,043	1427	Database	United States	1, 2, 11, 27	8
Chang 2021	1996–2013	Co	48,828	692	Database	Taiwan	1, 2, 11, 14, 15, 26	8
Palermo 2021	2015–2017	Co	104	104	Database	Italy	1, 2, 19, 25	7
Kim 2022	2002–2015	C‐C	15,130	3026	Database	South Korea	1, 2, 4, 7, 22	8
Nguyen 2023	2004–2018	Co	73,925	693	Database	France	4, 5, 7, 8, 18, 20, 23	8
Lin 2021	1996–2008	RCT	77	77	N/A	Taiwan		N/A
Stevens 2022	2016–2020	RCT	235	235	N/A	United Kingdom		N/A

*Note*: 1: age, 2: sex, 3: race, 4: residency, 5: education, 6: socioeconomic status, 7: smoking, 8: body mass index, 9: Charlson index, 10: chronic obstructive pulmonary disease, 11: cardiovascular diseases, 12: dementia, 13: duration of hypercholesterolemia, 14: hypertension, 15: CVA, 16. diabetes, 17: depression, 18: comorbidities, 19: use of medication (lipophilic or hydrophilic statins, fibrates, antihypertensive, antidiabetics, antiplatelets, nonsteroidal anti‐inflammatory drugs, antipsychotics, and antidepressants) 20: caffeine, 21: lactose, 22: alcohol, 23: physical activity, 24: low‐density lipoprotein cholesterol, 25: average total cholesterol, 26: hyperlipidemia, 27: disease duration.

### 3.4 Meta‐analysis results for the first research question

#### 3.4.1 The association between statin use and PD Risk

**FIGURE 2:**
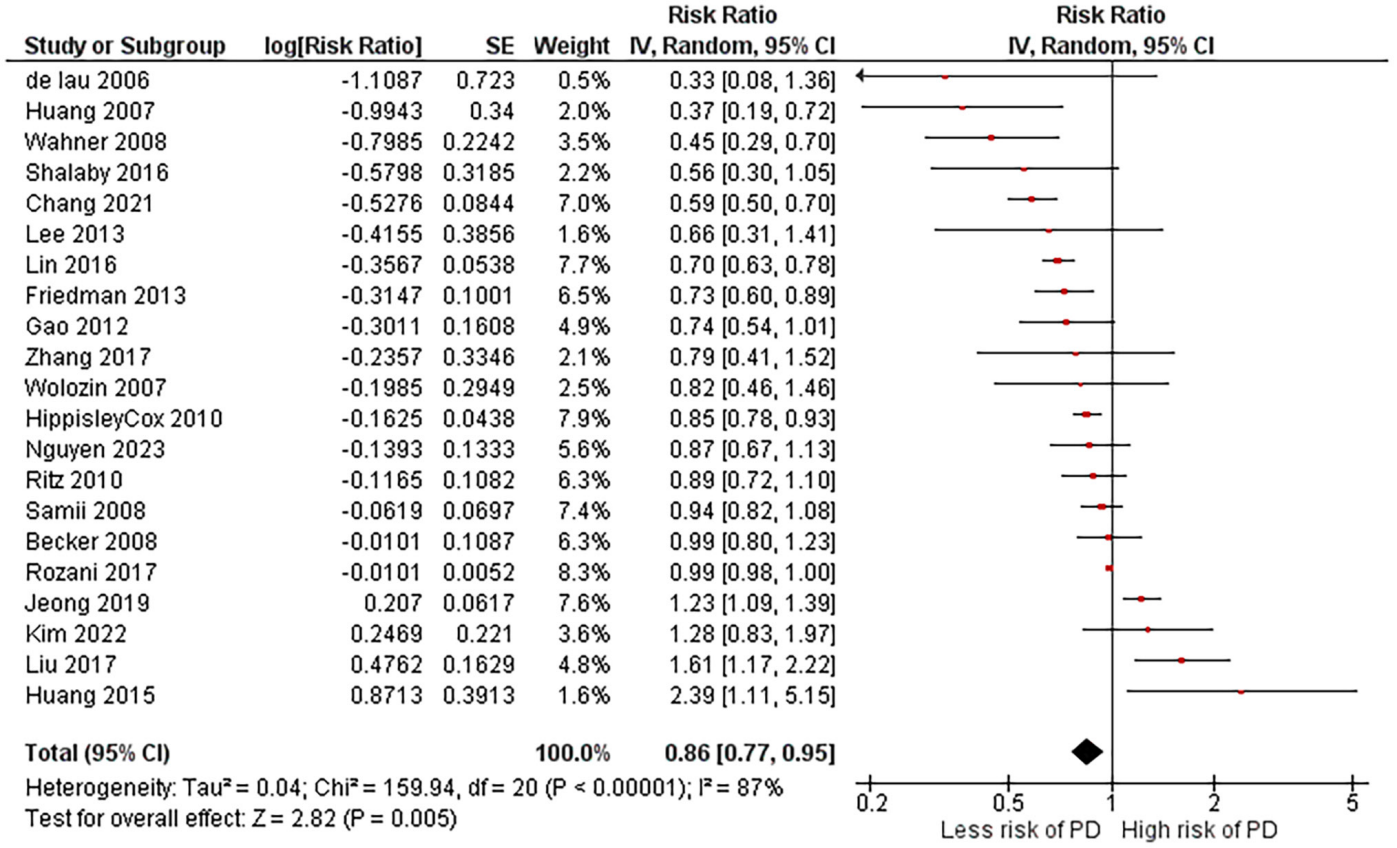
Main meta‐analysis results for the first research question (the association between statins and PD risk).

The pooled RR from 21 studies showed a significant association between statin use and a reduced risk of PD. Individuals with a history of statin use had lower risk of developing PD (RR 0.86, 95% CI (0.77–0.95), *p* < 0.005) (Figure 2). The random‐effect model was used because of the significant heterogeneity among studies (*χ* ^2^ *p* < 0.00001; *I* ^2^ = 87%).

#### 3.4.2 Sensitivity analysis for PD risk

We conducted a sensitivity analysis for the PD risk outcome in multiple scenarios by excluding one study in each scenario and repeated this process 21 times. This method is used to investigate whether a particular study is accounting for the underlying high heterogeneity. We found that heterogeneity persisted in every scenario meaning that heterogeneity cannot be attributed to a single study but larger variations between the studies.

#### 3.4.3 Subgroup analysis for PD risk

We tried to explain the heterogeneity by conducting subgroup analysis according to six different factors (namely, study design, location, adjustment for age, adjustment for sex, adjustment for smoking, and statin type) as presented in Table 2. This method failed to explain the heterogeneity as the heterogeneity persisted in most of the study groups except for the following groups: The studies conducted in Europe (*χ* ^2^ *p* = 0.47, RR 0.87, 95% CI [0.81–0.93], *p* < 0.001) and the studies that did not adjust for age (*χ* ^2^ *p* = 0.66, RR 0.87, 95% CI [0.82–0.93], *p* < 0.001). This means that the heterogeneity could not be completely explained by variation in any of these factors but likely by deeper differences in the study methodology.

**TABLE 2:** The results of subgroup analyses based on various factors.

	Number	OR (95% Cl)	*p* value	Chi‐squared *p* value, *I* ^2^
All studies	21	0.86 (0.77, 0.95)	0.005	<0.001, 87%
Study design				
Cohort	12	0.84 (0.73, 0.96)	0.01	<0.001, 91%
Case–control	9	0.86 (0.69, 1.08)	0.19	<0.001, 77%
Region				
North America	10	0.87 (0.68, 1.12)	0.28	<0.001, 83%
Europe	5	0.87 (0.81, 0.93)	<0.001	0.47, 0%
Asia	6	0.79 (0.62,1.01)	0.06	<0.001, 94%
Adjusted for age				
Yes	17	0.84 (0.73, 0.95)	0.008	<0.001, 90%
No	4	0.94 (0.84, 1.04)	0.21	0.84, 0%
Adjusted for sex				
Yes	14	0.84 (0.71, 0.99)	0.04	<0.001, 91%
No	7	0.87 (0.82, 0.93)	<0.001	0.66, 0%
Adjusted for smoking				
Yes	12	0.84 (0.70, 1.00)	0.05	<0.001, 83%
No	9	0.86 (0.72, 1.03)	0.11	<0.001, 91%
Statin type				
Simvastatin	6	0.64 (0.48, 0.86)	0.003	<0.001, 94%
Atorvastatin	6	0.69 (0.53, 0.90)	0.006	<0.001, 83%
Lovastatin	3	1.00 (0.81, 1.24)	0.98	0.04, 70%
Pravastatin	3	1.34 (0.58, 3.09)	0.49	0.04, 68%

The effect estimate continued to show statistical significance in cohort studies, the studies conducted in Europe, the studies conducted in Asia, the studies that adjusted for age, the studies that adjusted for sex and the studies that did not, and the studies that used simvastatin and atorvastatin. In the remaining groups, the result was not statistically significant.

#### 3.4.4 Publication bias for PD risk

**FIGURE 3:**
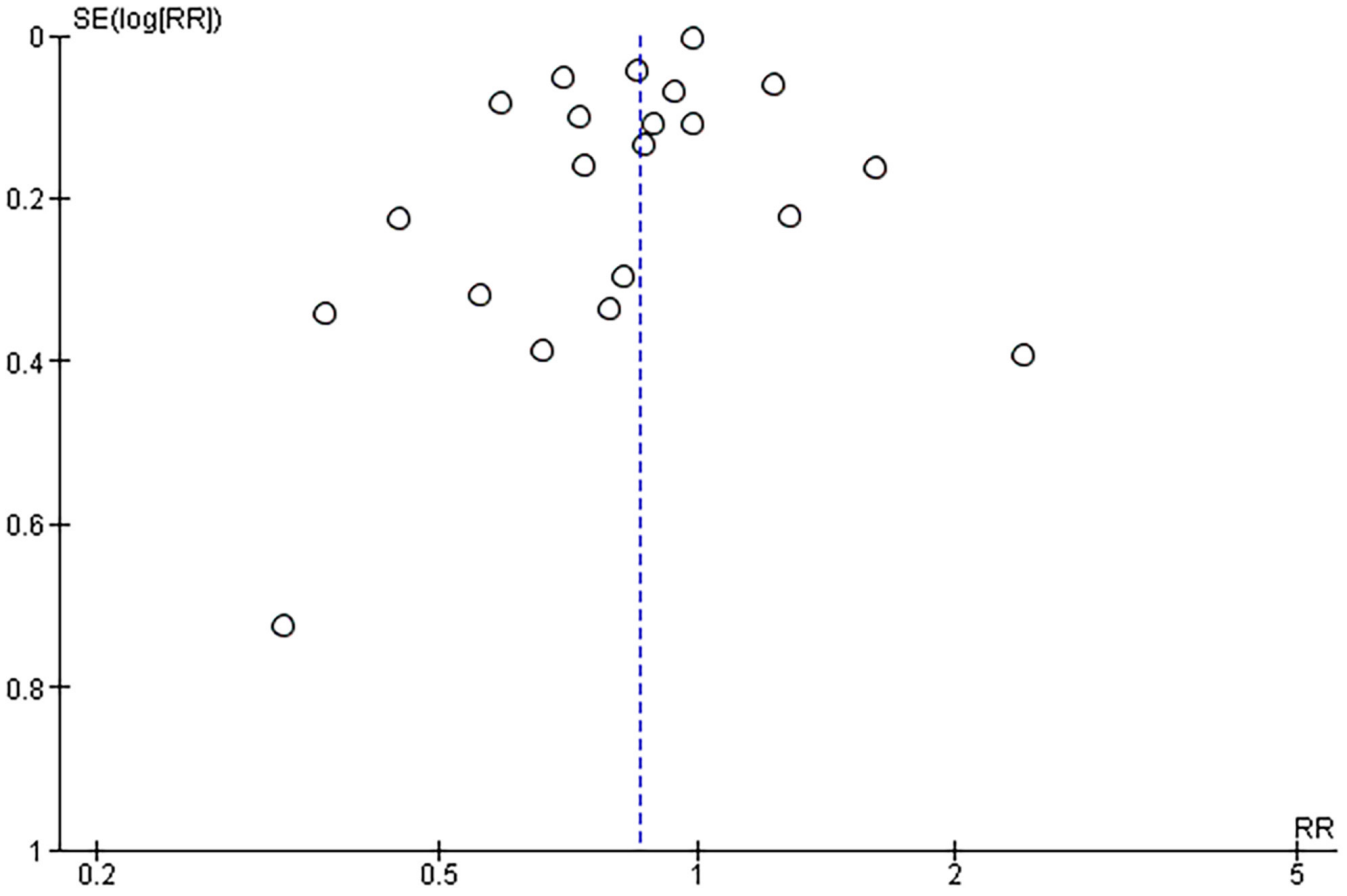
Funnel plot for assessing publication bias for the first research question (the association between statins and PD risk).

In accordance with the prespecified criteria described above in the methods section (see 2.8. publication bias), the number of the included studies in the meta‐analysis model of the first research question (>10) allowed for publication bias assessment using the funnel plot. The visual interpretation of the funnel plot (Figure 3) showed a symmetrical scatter of points on both sides of the mean effect size. This suggested a wide distribution of the study variabilities.

### 3.5 Meta‐analysis results for the second research question

#### 3.5.1 The pooled change in UPDRS III

**FIGURE 4:**
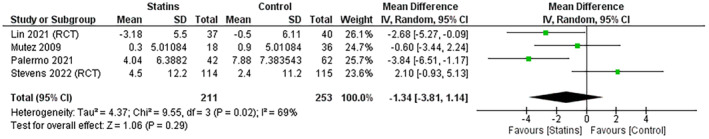
Meta‐analysis results of the second research question (the effect of statins on PD symptoms progression).

The pooled meta‐analysis did not find a significant difference in UPDRS III scores between the statins and control groups (MD −1.34 points, 95% CI [−3.81 to 1.14], *p* = 0.29, Figure 4). This analysis was based on four studies (two observational and two RCTs) with a total of 464 participants (statins: *n* = 211 patients and placebo: *n* = 253 patients). However, the studies included in the analysis were not homogeneous as indicated by the significant chi‐squared *p*‐value and I‐squared value (*p* = 0.02, *I* ^2^ = 69%).

#### 3.5.2 Subgroup analysis

We run a subgroup analysis according to the study design. For the subgroup of cohort studies, the overall MD in UPDRS‐III between statins and control did not favor either group (MD −2.26 points, 95% CI [−5.43 to 0.92], *p* = 0.16). Similarly, for the subgroup of RCTs, the overall MD in UPDRS‐III between statins and placebo did not favor either group, with a nonsignificant MD of −0.36 (95% CI [−5.04 to 4.32], *p* = 0.88).

#### 3.5.3 Sensitivity analysis

**FIGURE 5:**
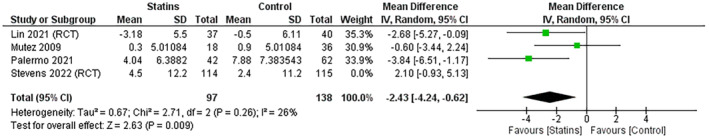
Sensitivity analysis results after removing the Stevens 2022 study.

We conducted a sensitivity analysis by excluding each individual study at a time. Excluding the study of Stevens 2022^8^ from the meta‐analysis model resolved the heterogeneity (*χ* ^2^ *p* = 0.26, *I* ^2^ = 26%) as presented in Figure 5. After removing this study, the overall MD between statin and placebo favored statins, with a statistically significant MD of −2.43 points (95% CI [−4.24 to −0.62], *p* = 0.009). This difference could be explained by the higher baseline UPDRS‐III score compared to the other studies.

#### 3.5.4 Publication bias

Based on the criterion descripted above in the Methods section, the assessment of publication bias for the second research question was not reliable because the number of included studies was insufficient.^43^ Therefore, we did not run the publication bias for this meta‐analysis model.

#### 3.5.5 GRADE assessment of the certainty in evidence

Based on GRADE assessment, the quality of evidence on the changes in UPDRS‐III score (MD −1.34 points with 95% CI [−3.81 to 1.14]) was evaluated as “low.” The quality of evidence is limited by the observational design of two studies, which lacks random allocation. There is also a significant heterogeneity in the pooled estimate of UPDRS‐III score (Table 3). Therefore, the evidence is downgraded by two levels from the initial evaluation “moderate.” The “low” assessment of evidence means that the true effect might be markedly different from the estimated effect. Therefore, future studies are needed to increase our confidence in the estimate.

## 4 DISCUSSION

### 4.1 Summary of the key findings

Our study revealed that individuals with a history of statin use have less risk of developing PD compared to those who do not have a history of statin use. However, statin supplementation for PD patients did not slow PD progression or improve motor functions. Our meta‐analysis does not support the use of statin for PD patients.

### 4.2 Explanation of the study findings

For the first research question, the subgroup analysis according to individual statins showed statistically significant results for simvastatin and atorvastatin as opposed to other statins (pravastatin and lovastatin). Lipophilicity is an important characteristic of drugs that enables them to cross the blood–brain barrier (BBB) and allows their central nervous system action. Lipophilic statins like simvastatin and atorvastatin could cross the BBB, which can help explain our findings. When comparing simvastatin to atorvastatin, our subgroup analysis shows that simvastatin was more protective than atorvastatin which confirms the results of previous studies that simvastatin is superior to atorvastatin at crossing the BBB.^44^

In the second research question, the only study that exclusively used simvastatin^8^ was the only study that reported an increased, rather than decreased, rate of symptom progression of PD with statin use. Excluding this study in the sensitivity analysis resolved the heterogeneity and resulted in statistically significant positive results. This contradicts the findings about simvastatin superiority from the first question subgroup analysis. These apparently conflicting results about individual statin difference in neuroprotection warrants future well‐designed studies with extensive subgroup analysis according to individual statins.

Another factor that explains the resolved heterogeneity after excluding Stevens 2022 is the higher baseline MDS‐UPDRS‐III score in this study compared to the other studies, meaning that this study included individuals with more severe disease compared to other studies. In disease modification trials, it is understood that the biological microenvironment of patients with advanced disease stage might be less viable to disease modification interventions and therefore, they may achieve less benefit. Within the context of this meta‐analysis, it is hard to establish this association given the limited number of included studies and the unavailability of individual patient data. Because Stevens 2022 is the largest included RCT in terms of sample size, excluding this study in sensitivity analyses reduced the overall statistical power and overall precision of the meta‐analysis.

### 4.3 Previous systematic reviews

Our results were consistent with a previous study conducted by Wu et al.,^13^ which suggested that statins may be beneficial for reducing the risk of PD. Both our study (OR = 0.86, 95% CI [0.77–0.95]) and Wu et al.'s (RR 0.79, 95% CI [0.68–0.91]) demonstrated that statins reduced the risk of PD incidence.

Compared to Wu et al. our review incorporated seven new studies, discussing the protective effect of statins on PD patients. Moreover, our study searched into additional areas beyond the reduction of PD risk. We investigated the potential of statins as a disease‐modifying agent to hinder motor symptom progression in patients with PD. We included four additional studies for this purpose.

### 4.4 Strengths and limitations

Regarding strengths, our study is the most comprehensive one on this topic (Table 4). It includes observational and interventional studies with 27 studies (9 case–controls, 16 cohorts, and 2 RCTs). We investigated different statins, including simvastatin, lovastatin, atorvastatin, and pravastatin, as a risk factor for developing PD and a disease‐modifying agent to improve the clinical outcomes in PD patients. Our meta‐analysis included more than 4 million participants from around the world. We performed broad subgroup and sensitivity analyses that would impact clinical decision‐making. Additionally, we think our study opens the door for more interventional studies considering the therapeutic effects of different statins for PD.

**TABLE 4:** Comparison with previous systematic reviews.

Study ID	Undela 2012	Bai 2016	Sheng 2016	Bykov 2017	Poly 2017	Yan 2019	Wu 2022	Our study
Number of studies (CC + cohort)	8 (5 + 3)	11 (5 + 6)	11 (5 + 6)	10 (4 + 6)	13 (6 + 7	17 (8 + 9)	18 (8 + 10)	27 (12 + 13 + 2 RCTs)
Sample size	1,472,938	3,513,209	2,787,249	Not reported	4,877,059	3,845,303	3.7 million	4,060,853
PD cases	15,102	21,011	18,316	Not reported	24,596	28,639	31,153	36,096
Combined effect estimate for PD risk	RR 0.77, 95% CI (0.64–0.92)	RR 0.81, 95% CI (0.71–0.92)	RR 0.74, 95% CI (0.62–0.90)	RR 0.75, 95% CI (0.60–0.92)	RR 0.70, 95% CI (0.58–0.84)	OR 0.92, 95% CI (0.86–0.99)	RR 0.79, 95% CI (0.68–0.9)	RR 0.86, 95% CI (0.77–0.95)
Subgroup analyses performed	Study design, quality, age adjustment, smoking adjustment, duration, and individual statin	Study design, quality, region, age adjustment, gender adjustment, and individual statin	Study design, quality, region, age adjustment, and confounders adjustments >3	Cholesterol adjustment (and study design subgroup analysis within it)	Study design, quality, region, age adjustment, gender adjustment, smoking adjustment, duration, and individual statin	Study design, quality, race, individual statin, and duration	Study design, quality, region, age adjustment, gender adjustment, smoking adjustment, duration, and individual statin	Study design, region, age adjustment, gender adjustment, smoking adjustment, and individual statin

**TABLE 3:** GRADE assessment of the certainty in evidence.

Outcome: mean change in UPDRS‐III score; estimate: MD −1.34 points with 95% CI (−3.81–1.14); studies: four studies (*n* = 464 participants)						
Risk of bias	Inconsistencies	Indirectness	Imprecision	Publication bias	Others	Final assessment
Downgrade by one level^a^	Downgrade by one level^b^	No	No	N/A	No	Low

Abbreviation: N/A, not applicable because of the small number of included studies (Egger et al).

^a^ Owing to the potential risk of bias in addition to the inclusion of non‐RCT studies.

^b^ Owing to the significant heterogeneity in the effect estimate (*I* ^2^ = 69%).

Limitations in our study included the significant heterogeneity among the studies in the main and subgroup analyses, which we tried to resolve by performing sensitivity and subgroup analyses. The variability between the study designs, durations, participants' characteristics, and outcome measures justifies this heterogeneity. Second, our study could not conclude the effect of different doses of statins on the outcomes of interest because of inadequate data. Third, most of the included studies (*n* = 23) were observational studies. Therefore, there might be several potentially confounding factors to consider.

Finally, it should be mentioned that mixing data from both observational studies and RCTs in the same meta‐analysis (the second research question in our review) is controversial as it may introduce bias to the results because of the inherent difference in observational and interventional studies. However, according to Borenstein et al.,^45^ observational studies and RCTs might be put together if they do not disagree with each other and are believed to address a common question. Moreover, we conducted subgroup analysis according to the study design. Both groups (observational group and RCT group) showed consistent overall effect size in the same direction which further justifies the combining of both designs.

### 4.5 Authors' conclusion

Although epidemiological observational studies showed that statin use was associated with a reduced risk of PD occurrence, current evidence is insufficient to support the role of statins in slowing the progression of PD. These findings are limited by the fact that most of the included studies are observational which carry a high risk of confounding bias. Therefore, future well‐designed RCTs are recommended.

## AUTHOR CONTRIBUTIONS

*Study conceptualization and design*: Ahmed Negida and Abdelrahman Mady. *Protocol design*: Abdelrahman Mady. *Abstract screening on Rayyan*, *full‐text screening and study selection*, *data extraction*, *and quality assessment*: All authors participated in these steps. *Data analysis*: Asma Daoud, Abdelrahman Mady, Menna Marwan, and Moaz Elsayed. *Writing introduction and conclusion*: Abdulrahman Krayim and Maickel Abdelmeseh. *Writing methods*: Abdelrahman Mady and Moaz Elsayed. *Writing results*: Abdelrahman Mady, Asmaa Alnajjar, and Taleb Alsalloum. *Writing discussion*: Abdelrahman Mady, Maickel AbdelMeseh, Menna Marwan, Asmaa Alnajjar, Taleb, Mohamed Alaa, and Nagham Abdalla. *Proofreading the manuscript*: Ahmed Negida. *Supervision and revision*: Matthew J. Barrett, Ahmed Negida, and Brian Berman.

## CONFLICT OF INTEREST STATEMENT

The authors have no conflict of interest to declare.

## Data Availability

The data that support the findings of this study are available from the corresponding author upon reasonable request.
